# One Carbon Metabolism and *S*-Adenosylmethionine in Non-Alcoholic Fatty Liver Disease Pathogenesis and Subtypes

**DOI:** 10.3390/livers2040020

**Published:** 2022-10-04

**Authors:** David Fernández-Ramos, Fernando Lopitz-Otsoa, Oscar Millet, Cristina Alonso, Shelly C. Lu, José M. Mato

**Affiliations:** 1 Precision Medicine and Metabolism Laboratory, CIC bioGUNE, BRTA, CIBERehd, Technology Park of Bizkaia, 48160 Derio, Bizkaia, Spain; 2 OWL Metabolomics, Technology Park of Bizkaia, 48160 Derio, Bizkaia, Spain; 3 Karsh Division of Gastroenterology and Hepatology, Cedars-Sinai Medical Center, Los Angeles, CA 90048, USA

**Keywords:** lipidomics, non-alcoholic fatty liver disease subtypes, *S*-adenosylmethionine

## Abstract

One carbon metabolism (1CM) can be defined as the transfer of a carbon unit from one metabolite to another and its replenishment by different sources of labile methyl-group nutrients: primarily choline, methionine, betaine, and serine. This flow of carbon units allows the biosynthesis of nucleotides, amino acids, formylated methionyl-tRNA, polyamines, glutathione, phospholipids, detoxification reactions, maintenance of the redox status and the concentration of NAD, and methylation reactions including epigenetic modifications. That is, 1CM functions as a nutrient sensor and integrator of cellular metabolism. A critical process in 1CM is the synthesis of *S*-adenosylmethionine (SAMe), the source of essentially all the hundreds of millions of daily methyl transfer reactions in a cell. This versatility of SAMe imposes a tight control in its synthesis and catabolism. Much of our knowledge concerning 1CM has been gained from studies in the production and prevention of nonalcoholic fatty liver disease (NAFLD). Here, we discuss in detail the function of the most important enzymes for their quantitative contribution to maintaining the flux of carbon units through 1CM in the liver and discuss how alterations in their enzymatic activity contribute to the development of NAFLD. Next, we discuss NAFLD subtypes based on serum lipidomic profiles with different risk of cardiovascular disease. Among the latter, we highlight the so-called subtype A for its serum lipidomic profile phenocopying that of mice deficient in SAMe synthesis and because its high frequency (about 50% of the NAFLD patients).

## 1. Impact Statement

Here, we discuss the critical role of *S*-adenosylmethionine (SAMe) in modulating liver lipid metabolism and how SAMe deficiency leads to liver fat accumulation and its progression to nonalcoholic steatohepatitis. Next, the subtypes of nonalcoholic fatty liver disease (NAFLD) based on serum lipidomic profiles with different risk of cardiovascular disease are discussed, highlighting the so-called subtype A for its serum lipidomic profile phenocopying that of mice deficient in SAMe synthesis and because of its high frequency.

## 2. Introduction

### 2.1. Metabolic Considerations for Nonalcoholic Fatty Liver Disease (NAFLD)

Monarch butterflies, salmons, bar-tailed geese, gray whales, arctic ground squirrels, and grizzly bears all have in common amassing massive reserves of fat in preparation for travelling long distances non-stop or to spend long periods of hibernation. The source of energy, the fuel to carry out these prodigious feats, are triglycerides (TG). TG are stored mainly in adipose tissue, which is specialized for storing fat as energy source, and to a lesser extent in the liver, where they are used to synthesize TG-rich, very low-density lipoproteins (VLDL), and they are eventually secreted into the circulatory system to provide TG and cholesterol to peripheral tissues. TG produce an amount of energy of 9 kcal per gram, like that of diesel or anthracite coal, and more than twice as much as burning carbohydrates. This is due to two factors: fatty acids (FA) are more chemically reduced than carbohydrates, and TG are stored in a nearly anhydrous state (5% water), whereas carbohydrates (stored as glycogen) have higher water content (around 70%). These two factors are probably why TG rather than glycogen evolved as the main energy store in living organisms.

Nonalcoholic fatty liver disease (NAFLD, also known as metabolic associated fatty liver disease [1]) represents a spectrum of diseases that occur in the absence of excessive alcohol consumption, ranging from the isolated accumulation of hepatic TG (steatosis, NAFL) to the appearance of inflammation and liver injury (non-alcoholic steatohepatitis, NASH) and eventually progressing to fibrosis/cirrhosis and hepatocellular carcinoma. NAFLD is a common condition strongly associated with the metabolic syndrome (obesity, insulin resistance/type 2 diabetes mellitus (T2D), high blood pressure, and dyslipidemia), which affects more than 25% of the adult population worldwide. The most common cause of death from NAFLD is cardiovascular disease (CVD) [2–6].

NAFLD arises when circulating FA uptake and the de novo lipogenesis (DNL) saturate the liver’s mitochondrial capacity to oxidize FA and its secretion as TG assembled into VLDL particles. The relationship between the intrahepatic TG (IHTG) content and FA oxidation or VLDL export is not linear. VLDL secretion, for example, is curvilinear and saturates when the average IHTG content is about 10% of its wet weight. Moreover, there are striking differences among individuals. The saturation of VLDL export at higher IHTG values is not only a physiological adaptation and evolutionary advantage in anticipating prolonged periods of food scarcity through hepatic TG accumulation but also serves to control the concentration of VLDL in circulation, whih is a critical factor, as high levels of VLDL often lead to elevated concentrations of low-density lipoproteins (LDL), which are a major risk factor for CVD [2–6].

In addition to increasing food intake, an evolutionary strategy for hepatic fat accumulation in preparation for periods of food deprivation may be the timing between the increased exposure to low-quality diets deficient in certain essential nutrients, such as methionine and choline, and the mobilization of FA from adipose tissue to the liver. In the 1930s, Charles Best found that the accumulation of fat in the liver of rats and dogs, which occurs under certain experimental conditions, could be effectively counteracted by the administration of choline, a phenomenon known as “lipotropism” [7–10]. In a later study, Tucker and Eckstein discovered the lipotropic action of methionine; and in 1941, Vincent Du Vigneaud discovered transmethylation (also known as methylation), that is, the addition of a methyl group from methionine to a substrate and its relationship to the biosynthesis of choline and other methylated metabolites such as creatine and creatinine [7–10]. Du Vigneaud also discovered transsulfuration, the metabolic pathway that involves the transfer of the sulfur atom from methionine to serine for the synthesis of cysteine and glutathione (GSH, the most abundant cellular antioxidant) [7–10]. The most transcendental discovery was made by Giulio Cantoni in 1953 when he showed that, to transfer its methyl group, methionine first needs to be converted to a sulfonium ion by reacting with ATP to form *S*-adenosylmethionine (SAMe, also known as AdoMet) [11]. In 2001, we discovered that mice deficient in hepatic SAMe synthesis are more susceptible to choline-deficient diet-induced fatty liver injury and spontaneously developed steatohepatitis [12]; and in 2017, Alonso et al. found that SAMe-deficient mice that were administered SAMe orally showed a reduction of liver lipid accumulation, a decrease of liver inflammation and fibrosis, and a reduction in serum transaminases [13]. Altogether, these findings indicate that SAMe is a critical regulator of liver lipid homeostasis and support the idea that a reduction in SAMe facilitates the accumulation of hepatic TG in preparation for periods of food deprivation. Moreover, these results raise the question of how SAMe regulates liver lipid metabolism and whether SAMe deficiency is frequent in NAFLD patients.

### 2.2. SAMe and One-Carbon Metabolism in Healthy Liver

**Figure 1.**
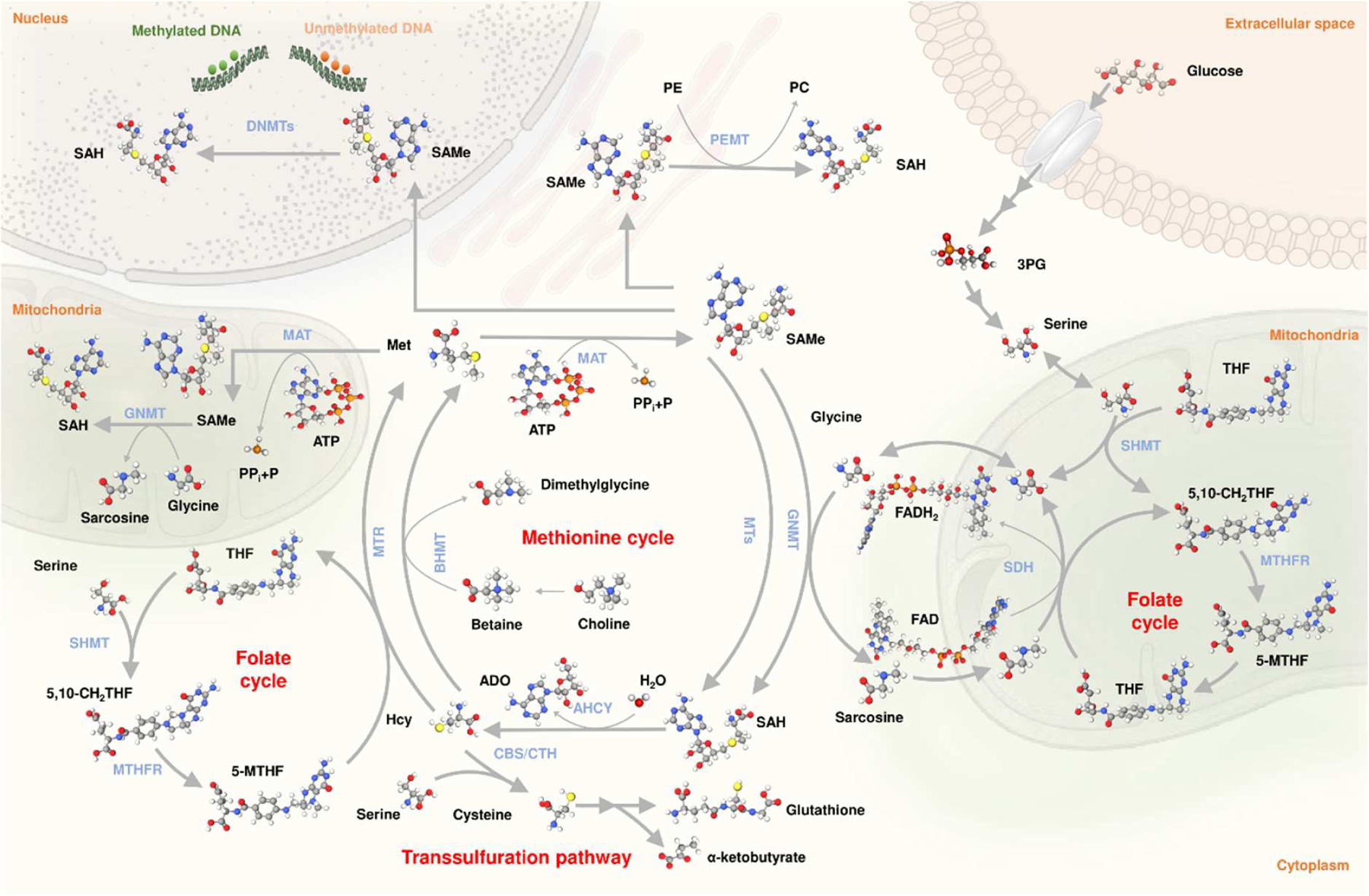
One-carbon metabolism. Representation of the main reactions involved in one carbon metabolism into the cytoplasm, nucleus, and mitochondria. Represented reactions include methionine cycle, folate cycle, transsulfuration pathway, PEMT flux, and DNA methylations, connected to glycolysis via 3PG. ADO, adenosine, AHCY, *S*-adenosylhomocysteinase; ATP, adenosine triphosphate; BHMT, betaine-homocysteine methyltransferase; CBS, cystathionine β-synthase; CTH, γ-cystathionine lyase; DNMTs, DNA methyltransferases; FAD, Flavin adenine dinucleotide; FADH2, reduced Flavin adenine dinucleotide; GNMT, glycine-*N*-methyltetrahydrofolate; 5,10-CH_2_THF, 5,10-methylenetetrahydrofolate; MTs, methyltransferases; MTR, MTHF-homocysteine methyltransferase; MTHFR, 5,10-methylenetetrahydrofolate reductase; P, phosphate; PC, phosphatidylcholine; PE, phosphatidylethanolamine; PEMT, phosphatidylethanolamine *N*-methyltransferase; 3PG, 3-phosphogycerate; PPi, pyrophgosphate; SAH, *S*-adenosylhomocysteine; SAMe, *S*-adenosylmethionine; SDH, sarcosine dehydrogenase; SHMT, serine hydroxymethyltransferase; THF, tetrahydrofolate.

The enzyme catalyzing the synthesis of SAMe from methionine and ATP is called methionine adenosyltransferase (MAT) [11] (Figure 1). In mammals, there are two genes encoding MATs: *MAT1A* and *MAT2A*. The protein encoded by *MAT1A*, MATα1, is found as a homotetramer (MATI) and a homodimer (MATIII), whereas the protein encoded by the *MAT2A* gene, MATα2, is found forming a homotetramer (MATII) [10,14–16]. While *MAT1A* is expressed mainly in the adult liver, *MAT2A* is expressed in all extrahepatic tissues and fetal liver [10,14–16]. Though SAMe can be synthesized in vitro by MATII alone, MATα2 can form a hexameric complex with MATβ (4MATα2 and 2MATβ subunits) [17], a protein encoded by *MAT2B* that stabilizes and regulates the catalytic activity of MATII [17,18] (Bailey 2021). This raises the question of whether MATII exists without being associated to MATβ under physiological conditions. The combination of MATI (low K_m_ and V_max_) and MATIII (high K_m_ and V_max_) provide the liver with the ability to efficiently catabolize methionine in the blood and maintain the concentration of this amino acid under control [9,10,16]. Accordingly, about 50% of the methionine in the diet is metabolized by the liver [19,20]. In contrast, the synthesis of SAMe by extrahepatic cells, which express MATII, an enzyme with low K_m_ and V_max_, has as its main objective to satisfy local methylation needs.

When dietary intake of labile methyl groups (methionine, choline, betaine, serine) exceeds what is needed for ongoing methylation reactions, such as that produced in the liver after eating a protein-rich meal, and to avoid the undesirable methylation of DNA, proteins, phospholipids, etc., methyl balance is maintained by the elimination of excess SAMe by the enzyme glycine *N*-methyltransferase (GNMT) (Figure 1), which is expressed primarily in the liver [7,21]. GNMT catalyzes the conversion of SAMe and glycine to *N*-methylglycine (also called sarcosine) and *S*-adenosylhomocysteine (SAH), which is reversibly hydrolyzed to homocysteine and adenosine by the enzyme *S*-adenosylhomocysteinase (AHCY). In turn, sarcosine dehydrogenase (SDH), an enzyme localized in the mitochondrial matrix, catalyzes the oxidative demethylation of sarcosine, regenerating glycine with the formation of formaldehyde. The demethylation of sarcosine can proceed in the presence or absence of tetrahydrofolate (THF). In the presence of THF, SDH catalyzes the condensation of THF with formaldehyde to form 5,10-methylene-THF. Methylene-tetrahydrofolate reductase (MTHFR), in turn, converts 5,10-methylene-THF to 5-methyl-THF (5-MTHF), which is used by the enzyme MTHF-homocysteine methyltransferase (MTR, also called methionine synthase) to remethylate homocysteine forming methionine and THF (Figure 1), a process referred as methylneogenesis. 5,10-methylene-THF can also be synthesized by serine hydroxymethyltransferase (SHMT), the enzyme that catalyzes the reversible reaction of serine and THF to form glycine and 5,10-methylene-THF. Additionally, homocysteine remethylation can be achieved by betaine-homocysteine *S*-methyltransferase (BHMT), the enzyme that catalyzes the transfer of a methyl group of betaine (also called *N*,*N*,*N*-trimethylglycine, a product of choline oxidation) to homocysteine to produce methionine and *N*,*N*-dimethylglycine (Figure 1). While MTR is expressed in all mammalian cells and tissues including the liver, BHMT is mainly expressed in the liver. The combination of MTR (low K_m_ for homocysteine) and BHMT (high K_m_ for homocysteine) provide the liver with the capacity to efficiently maintain the physiological concentration of homocysteine. This is of great physiological relevance since homocysteine is in equilibrium with SAH (AHCY is the only reversible enzyme of the methionine cycle), a potent inhibitor of methylation reactions (the SAMe/SAH molar ratio is an indicator of a cell’s methylation capacity). Around one-half of the liver homocysteine is metabolized by the combination of MTR and BHMT and the other half by the transsulfuration pathway, the route involving the transfer of the sulfur atom from methionine, via homocysteine, for the synthesis of cysteine, taurine, and GSH, the major pathway for the metabolism of the sulfur-containing amino acids in mammals (Figure 1). The transsulfuration pathway involves the transfer of the sulfur atom of homocysteine to serine to yield cysteine, a two-step process mediated by two different enzymes, cystathionine β-synthase (CBS) and γ-cystathionine lyase (CTH), which is followed by the biosynthesis of γ-glutamylcysteine from glutamate and cysteine, and finally, the formation of GSH—the main cellular antioxidant—from γ-glutamylcysteine and glycine [22]. SAMe inhibits MTHFR and BHMT, and activates GNMT and CBS, whereas MTHF inhibits GNMT. These allosteric interactions together with the existence of GNMT play a central function in maintaining methyl balance regardless of fluctuations in the input of methionine, choline, betaine, and serine [23]. In addition to GNMT, the most important quantitative contributors to hepatic SAMe-dependent methylations are the synthesis of phosphatidylcholine (PC) by three successive *N*-methylations are the synthesis of phosphatidylcholine (PC) by three successive *N*-methylations of PE by the enzyme PE *N*-methyltransferase (PEMT) (Figure 1), and the synthesis of creatinine by guanidinoacetate *N*-methyltransferase (GAMT) [20].

SDH and MTR connect the methionine and folate cycles forming a metabolic network of highly interconnected and compartmentalized reactions known as one-carbon metabolism (1CM) (Figure 1). 1CM is also connected to glycolysis via 3-phosphogycerate, whose catabolism can be diverted from glycolysis toward serine synthesis through a pathway involving three enzymes: phosphoglycerate dehydrogenase, phosphoserine amino-transferase, and phosphoserine phosphatase [24]. In sum, 1CM can be defined as the transfer of a carbon unit from one metabolite to another and its replenishment by different sources of labile methyl-group nutrients (primarily choline, methionine, betaine, and serine). This flow of carbon units allows the biosynthesis of nucleotides, amino acids, formylated methionyl-tRNA, polyamines, GSH, phospholipids, detoxification reactions, maintenance of the redox status and the concentration of NAD, and methylation reactions including epigenetic modifications. That is, 1CM functions as a nutrient sensor and integrator of cellular metabolism [25]. This concept is evidenced in Kwashiorkor, a syndrome seen in children continuously fed poor-quality diets, where the development of hepatic steatosis and reduced plasma TG is associated with low levels of serum methionine and related 1CM metabolites, such as choline and homocysteine, and byproducts of the transmethylation (asymmetric dimethylarginine) and transsulfuration (cysteine and GSH) pathways [26]. The importance of 1CM is also underlined by the fact that antifolate chemotherapy is widely used since its discovery in 1948 [27], which further points to the importance to maintain the right balance between methylneogenesis and SAMe-dependent methylations.

Recently, our group reported both MATA1 and GNMT are found within the mitochondria of hepatocytes, where they are important in maintaining normal mitochondrial function [28,29]. Both MAT1A and GNMT are known to be downregulated in more advanced human NAFLD [30]. Although GNMT was found to be critical for Complex II activity [29], we speculate they may also be regulating the methylation capacity within the mitochondria.

### 2.3. SAMe and 1CM in NAFLD

Much of our knowledge concerning 1CM has been gained from studies in the production and prevention of fatty livers. As mentioned above, the connection between 1CM, particularly methionine and choline, and the accumulation of hepatic fat (steatosis) has been recognized for 90 years [8–10]. Since then, dietary choline deficiency has been frequently used to develop animal models of NAFLD [31–37]. It was later found that a greater depletion of the intracellular methyl-group pool, including the content of hepatic SAMe [38,39], could be attained by feeding mice or rats a diet deficient both in methionine and choline (MCD diet), leading to the development of NASH [31–37]. Mice fed a MCD diet show impaired export of VLDL, which is the main cause of the accumulation of hepatic fat in this model [40]. The MCD diet model of NASH, although different from human NASH in terms of having weight loss and low blood TG and glucose levels, induces a liver disease that is histologically similar to human NASH and has proven useful in studying the mechanism of injury in steatohepatitis and fibrosis [40,41] and to demonstrate the pre-clinical efficacy of pharmacologic therapies for NASH, such as Aramchol [42].

Of the eleven genes most important for their quantitative contribution to maintain the flow of carbon units through 1CM in the liver (*MAT1A, AMD1, GNMT, PEMT, GAMT, AHCY, CBS, BHMT, MTR, MTHFR,* and *SHMT1/2*), the mRNA content of six of them—catalyzing SAMe synthesis (*MAT1A*) and catabolism (*GNMT*), homocysteine synthesis (*AHCY*), and remethylation (*BHMT*, *MTHFR*)—and the first step in the transsulfuration pathway (*CBS*) is lower among obese NASH patients compared to individuals with metabolically healthy obesity (MHO), while obese NAFL subjects are intermediate between MHO and NASH patients [43,44]. Furthermore, hepatic SAMe content has been shown to be lower in NASH patients than in controls [45]. In addition, numerous studies indicate that the deletion of five of these genes (*Mat1a*, *Gnmt*, *Cbs*, *Bhmt*, and *Mthfr*) in mice leads to the spontaneous development of NAFLD. The homozygous deletion of the sixth of these genes, *Ahcy*, is embryonically lethal [46–48]. In humans, patients with *AHCY* deficiency display severe hepatic, muscular, and cognitive dysfunction, including multiorgan failure followed by death soon after birth [48,49].

#### 1. MAT1A

Deletion of *Mat1a* in mice fed a normal diet leads to the spontaneous development of steatosis that progresses to NASH, fibrosis [12,13], and eventually hepatocellular carcnoma [50]. *Mat1a* deletion in mice resulted in a reduction in hepatic SAMe level and in the content of downstream metabolites such as methylthioadenosine (MTA, a biomarker of polyamine synthesis) [51], PC(22:6) and PC(20:4) (biomarkers of the activity through the PEMT pathway), NAD (an essential coenzyme involved in redox reactions), DNA methylation (an epigenetic mechanism used by cells to control gene expression); hypotaurine, taurine, and GSH (biomarkers of the activity through the transsulfuration pathway); and reduced molar ratio of PC/PE (relatively small alterations in the PC/PE ratio can contribute to the development of NASH) [12,13,52]. *Mat1a* deletion also leads to the accumulation in the liver of methionine and upstream metabolites, such as serine (a labile methyl-group donor and GSH precursor), MTHF (an inhibitor of GNMT and indicator of the decoupling between the methionine and folate cycles), and PE(22:6) and PE(20:4) (substrates of the PEMT pathway) [12,13]. Furthermore, global quantitative proteomics identified six key functional processes altered in *Mat1a*-knockout (KO) mice, namely inhibition of FA metabolism, TCA cycle, peroxisomal protein import, pyruvate metabolism, and activation of GSH reactions and metabolism of xenobiotics by cytochrome P450 (CYP) [53]. *Mat1a*-KO mice showed impaired assembly and export to the blood of VLDL, which is probably the main cause of the accumulation of hepatic fat in this model [52]. Although liver PC is synthesized by both the PEMT and the CDP-choline (the transfer of phosphocholine to diacylglycerol via CDP-choline) pathways, both pathways are independently required for the normal incorporation of TG and CE into VLDL and their export to the blood [54,55]. The PEMT pathway, which is expressed mainly in the liver, accounts for about 30% of liver PC. Whereas the composition of PC synthesized by the PEMT pathway is rich in long-chain polyunsaturated fatty acids, such as docosahexaenoic acid (22:6) and arachidonic acid (20:4), PC molecules synthesized by the CDP-choline pathway are rich in long-chain, saturated, and monounsaturated fatty acids, such as palmitic acid (16:0), stearic acid (18:0), oleic acid (16:1), and linoleic acid (18:1) [55]. In addition, *Mat1a*-KO have reduced mitochondrial membrane potential and higher mitochondrial CYP2E1, which leads to higher levels of reactive oxygen species (ROS) [28,56]. MATA1 interacts with numerous liver proteins but with a preference of mitochondrial proteins, including CYP2E1 [28]. The interaction of MATA1 with CYP2E1 facilitates CYP2E1 methylation, leading to its degradation through the proteasomal pathway [28]. In sum, mice lacking *Mat1a* secrete less VLDL-TG and show impaired mitochondrial function, increased ROS, and reduced FA β-oxidation, GSH content, and PC/PE molar ratio and the development of NASH and fibrosis. Accordingly, treatment of 10-month-old *Mat1a*-KO mice for 2 months with SAMe restored normal liver histology [13].

#### 2. GNMT

Compared to wild-type (WT) animals, the absence of GNMT resulted in up to a 7-fold increase of methionine and up to a 35-fold increase of SAMe in the liver. The amount of SAH was decreased 3-fold in *Gnmt*-KO mice compared to WT, and the molar ratio SAMe/SAH increased from 3 in WT to 300 in KO mice [57]. The reduction in SAH could be explained by an increased flow of homocysteine (which is in chemical equilibrium with SAH) through the transsulfuration pathway triggered by the activation by SAMe of CBS. Compared to WT mice the liver of *Gnmt*-KO mice showed increased DNA, histone, and PE methylation [58] and augmented polyamine synthesis [58,59] and reduced gluconeogenesis [59]. Although the mechanism by which SAMe regulates the flux of pyruvate to glucose is not well-understood, it is important to note that glucose fuels the methionine cycle through the synthesis of one-carbon units from serine (Figure 1). Therefore, a reduction in gluconeogenesis could be a mechanism to counteract the metabolic changes induced by SAMe accumulation in *Gnmt*-KO mice, such as polyamine synthesis, a metabolic pathway essential for oncogenicity [60]. The increased flux from PE to PC results in a reduction in hepatic PE content and elevated PC/PE molar ratio. The excess of PC thus generated is catabolized, leading to TG synthesis and steatosis, via diacylglycerol, which further progresses to steatohepatitis, fibrosis, and hepatocellular carcinoma [58,61]. Collectively, these results demonstrate that SAMe regulates liver lipid metabolism through a concerted series of homeostatic actions that include reduction of the PC/PE molar ratio and inhibition of VLDL-TG secretion at low SAMe and increase of the PC/PE molar ratio and activation of TG synthesis via PEMT at high SAMe. In summary, it appears that there is an optimal range of SAMe concentration in the liver that is mainly maintained by the concerted activity of MATI/III and GNMT (SAMe is an allosteric regulator of GNMT activity), and that below or above this range, liver fat accumulates, which eventually may progress to NASH, fibrosis, and hepatocellular carcinoma.

#### 3. CBS

Homocysteine is a sulfur-containing non-protein amino acid found at a crossing point between the remethylation and the transsulfuration pathways. In the transsulfuration pathway, homocysteine condenses with serine to synthesize cystathionine in an irreversible reaction catalyzed by CBS that is activated by SAMe. Cystathionine is then hydrolyzed by CTH to form cysteine (which is used in redox control as a component of GSH for ATP production via hydrogen sulfide generation or catabolized to form pyruvate) and A-ketobutyrate, which is converted to succinyl-CoA, an intermediate in the TCA cycle [62]. CBS deficiency was first described by Mudd et al. [63] and is the most common inborn error of sulfur amino acids metabolism. Human CBS deficiency is characterized by elevations in plasma homocysteine and methionine [62]. Mouse models of CBS deficiency show increased liver homocysteine, SAMe, and SAH; and lower SAMe/SAH molar ratio, reduced liver GSH, and DNA hypomethylation [64,65]. Furthermore, CBS deficient mice have lower levels of PC, PEMT activity, and PC/PE molar ratio; developed steatosis; and showed mild liver injury [66].

#### 4. BHMT

Betaine is essential as a cellular osmolyte, as it maintains the integrity of cells by regulating the viscosity and ionic strength of the aqueous media, and as a source of labile methyl groups. In mammals, betaine is generated in two steps involving the mitochondrial enzymes choline dehydrogenase and betaine aldehyde dehydrogenase [67]. The primary control of this process is the transport of choline into the mitochondria since the exit of betaine from the mitochondria seems to be controlled by passive diffusion. BHMT catalyzes the transfer of a methyl group from betaine to homocysteine, forming *N*,*N*-dimethylglycine and methionine. *N*,*N*-dimethylglycine is further demethylated to be converted in sarcosine, which is also demethylated to yield glycine. BHMT is preferentially expressed in the liver, where it accounts for as much as 50% of the methylation of homocysteine. Accordingly, disruption of *Bhmt* in mice increases plasma and liver homocysteine levels, which in turn lowers liver SAMe content, increases SAH, and reduces the organ’s SAMe/SAH molar ratio. *Bhmt*-KO mice display fatty liver, which is due to a reduction in the export of VLDL associated with a marked decrease in total hepatic PC and ultimately hepatocellular carcinoma [68].

#### 5. MTHFR

MTHFR catalyzes the conversion of 5,10-methylene-THF to MTHF, a methyl donor for homocysteine remethylation to methionine. *MTHFR* is expressed in all mammalian tissues tested. Compared to WT mice, the absence of MTHFR resulted in an increase in serum homocysteine, a reduction of hepatic MTHF and SAMe, an increase in SAH, a reduction in the SAMe/SAH molar ratio and DNA hypomethylation, and slight microvesicular liver steatosis [69].

#### 6. PEMT

Disruption of *Pemt* in mice does not lead to the accumulation of either methionine or SAMe in the plasma or liver [20], which may be explained by the removal of excess SAMe by the synthesis of sarcosine via the GNMT pathway. *Pemt*-KO mice fed a control diet show no obvious changes in liver histology although hepatocytes from mice lacking PEMT secrete 50% less TG [70]. These results indicate that mitochondrial FA β-oxidation can compensate for the increase in hepatic TG in *Pemt*-KO mice. However, when fed a high-fat/high-cholesterol diet, the plasma of *Pemt*-KO mice contained less TG than wild-type (WT) mice fed the same diet due to reduced VLDL-TG secretion [54,70]. *Pemt*-KO mice fed a high-fat diet showed decreased PC/PE molar ratio and developed steatosis, which eventually progressed to NASH, faster than WT mice fed the same diet [54,70]. These results indicate that the PEMT pathway is important to maintain the liver’s full capacity to assemble and secrete VLDL-TG and that a deficiency in PE methylation could be a major metabolic restriction when hepatocytes are exposed to situations of metabolic stress, such as, for example, a diet high in fat or a diet deficient in choline.

Taken together, these studies demonstrate that there is an optimal range of SAMe and SAH concentrations in the liver that is maintained mainly by the concerted action of the enzymes MATI/III, GNMT, AHCY, CBS, BHMT, and MTHFR together with the adequate supply of labile methyl groups (methionine, choline, betaine, and serine) and that below and above this range of SAMe and SAH concentrations, fatty liver develops spontaneously, which can progress to NASH, fibrosis, and ultimately hepatocellular carcinoma.

### 2.4. Hepatic FA Metabolism

As mentioned above, liver FA come mainly from the blood, derived from TG lipolysis in adipose tissue and de novo synthesis from glucose. FA are then used for the synthesis of TG and other lipids, such as phospholipids (glycerophospholipids and sphingomyelins) and ceramides, or transported into the mitochondria to be oxidized. In humans, glucose is preferentially oxidized over FA to maintain blood glucose homeostasis [71]. A significant proportion of the TG in the liver are used to synthesize VLDL for export into the blood stream. Once in circulation, VLDL particles are converted to intermediate-density lipoproteins (IDL) and low-density lipoproteins (LDL) for delivery of FA to peripheral tissues. TG that are not secreted into the blood stream are stored in hepatocytes as lipid droplets. The accumulation of these lipid droplets in the liver is an essential feature of NAFLD [71,72]. Understanding how changes in hepatic lipid metabolism contribute to the development and progression of NAFLD is critical not only to understand its pathogenesis but also to understand the extent to which different mechanisms leading to the disease can contribute to the clinical manifestations of other pathologies associated to NAFLD, such as CVD and hepatocarcinogenesis.

Alterations in the main metabolic pathways that regulate FA homeostasis may vary between individuals, for example, by saturating the secretion rate of VLDL-TG at different IHTG concentrations [73] or by having different partition ratios between oxidation of carbohydrates over FA [74]. Most of the TG used for VLDL assembly and export are mobilized by lipolysis of pre-existing TG in the cytoplasm (which have been formed by DNL and esterification of the resulting FA), followed by their re-esterification, a process also known as TG remodeling [74]. This process separates liver TG synthesis and storage from TG secretion and regulates FA composition of circulating TG. Consistent with this, the FA composition of serum TG has been shown to differ between individuals with normal liver compared to NAFLD patients [72]. Furthermore, individuals with simple steatosis also show a different serum TG profile than NASH patients [75], an observation that has been used to develop non-invasive in vitro diagnostic tests of NAFLD [76,77]. In particular, two panels of body mass index (BMI)-stratified TG have been identified that discriminate between NAFLD and normal liver and between NASH and NAFL (Table 1) [77]. Importantly, in mice, these differences in serum TG profile have been shown to mirror differences in the liver composition of TG [13]. Differences in hepatic TG remodeling between individuals may be due to differences in genetic and environmental factors such as nutrition and lifestyle and could play a central function in the development and progression of NAFLD. The observation that the main genes associated with NAFLD (*PNPLA3, MBOAT7, HSD17B13,* and *TM6SF2*) often appear to have a central in lipid remodeling of hepatic TG and VLDL-TG export supports this concept [78,79]. The results from clinical trials showing limited benefit (20–50%) of the various treatments under study also support the existence of NAFLD patients with different metabolic phenotypes [4].

### 2.5. Lipidomics in NAFLD

Technical advances in high-performance liquid chromatography/mass spectrometry (LC/MS) have made it possible to obtain metabolic signatures from biological samples and to analyze their relationship with the disease [80]. In an analysis of serum lipidomes of a cohort of 535 patients (steatosis, *n* = 182; NASH, *n* = 183), our group identified a serum lipidomic signature associated with *Mat1a*-KO mice that subclassified this cohort into two main subtypes: a first subtype showing a serum lipidomic profile similar to that observed in *Mat1a*-KO mice (M-subtype) that was present in 49% of the patients and a second subtype showing a different profile (non-M subtype) [13]. In a subsequent study, we identified that a substantial proportion of the patients classified as non-M showed a lipidomic profile comparable to that observed in mice deficient in LDL receptor fed a high-fat diet (Ldlr-KO/HFD) [81], which is a mouse model of NASH showing increased VLDL secretion [82]. These results suggested that NAFLD patients show different VLDL-TG secretion rates (SR), being lower in patients with subtype M compared to NAFLD patients with non-M subtype, and that, consequently, both NAFLD subtypes could have different CVD risks.

**Figure 2.**
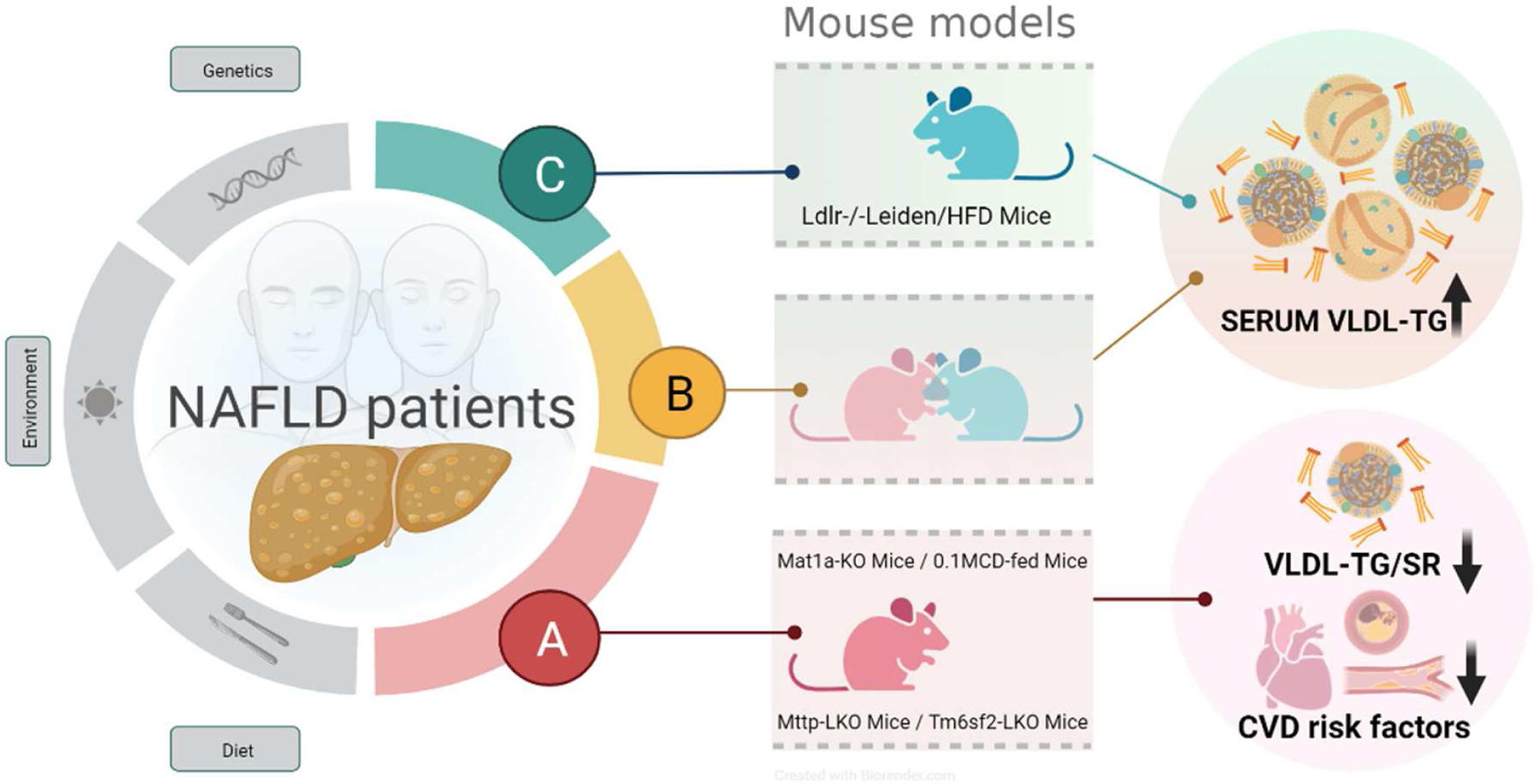
NAFLD patients subtypes in relation with murine models and CVD risk. Metabolomics analysis of serum allows classifying NAFLD patients into subtype A, B, or C by comparison with metabolomes of five mice models. Subtype A phenocopies the serum metabolome of four mice with impaired VLDL-TG secretion (Mat1a-KO, 0.1MCD, Mttp-LKO, Tm6sf2-+LKO), while subtype C phenocopies the metabolome of Ldlr−/−Leiden/HFD mice. Subtype B showed an intermediate signature. NAFLD subtypes align with CVD risk factors, with subtype A exhibiting a reduced CVD risk. Created with BioRender.com.

To confirm this hypothesis, we assembled a large cohort (*n* = 1154 individuals) of internationally recruited biopsied NAFLD patients from clinical centers in Israel, Europe (Czech Republic, Italy, UK, Spain), USA (Florida and California), and Chile. The comparison of the serum lipidome of this cohort of patients with NAFLD with the lipidomic signatures of four mouse models of NASH (*Mat1a*-KO, *Mttp*-KO, *Tm6sf2*-KO, and MCD diet) presenting reduced VLDL-TG SR and that of the *Ldlr*-KO/HFD mouse model resulted in the identification of three major metabolic subtypes (Figure 2) [83]. Confirming our previous findings [13], about half of the patients with NAFLD (541 patients, 47%) reproduced the metabolic features common to *Mat1a*-KO mice and the other three mouse models with impaired VLDL-TG-SR. This subgroup (formerly known as the M-subtype) was renamed as the A subtype [83]. Of the remaining patients, 308 (27%) of them showed a metabolomic phenotype similar to that of the *Ldlr*-KO/HFD mouse model and were classified as subtype C, and the rest of the NAFLD patients 305 (26%) were grouped together as subtype B [83]. A set of 12 lipid molecular species (9 PC, 2 sphingomyelins, and 1 TG) that discriminated among NAFLD subtypes A, B, and C was also identified (Table 2). In addition, a statistical model was generated for the categorization of patients with NAFLD into subtypes [83]. The serum lipoprotein profile (over 100 parameters analyzed [84]) of a subgroup of 197 samples from this cohort of NAFLD patients was also obtained. Compared to the A subtype, patients with NAFLD with subtype C exhibited hypertriglyceridemia, elevated VLDL-TG, VLDL-Apo-B, increased small and dense LDL particles (LDL_5_ and LDL_6_), and remnant lipoprotein cholesterol (the sum of cholesterol associated with VLDL an IDL). The concentration of these lipoproteins in NAFLD patients with B subtype were intermediate between those found in subtypes A and C [83]. These findings suggest that patients with subtype A show a favorable CVD risk profile compared to NAFLD patients with subtypes C and B. Accordingly, when the percentage of patients at high CVD risk was compared using the Framingham CVD risk score (FRS ≥ 15% at 10 years), patients with subtype B or C showed a higher CVD risk score (10% and 7%, respectively) than patients with subtype A (4%) [83]. The HOMA-IR index and HbA1c were similar among the three subtypes, and the use of lipid-lowering drugs was also similar between the different subtypes. Analysis of the association of four of the major known NAFLD genetic risk factors (*HSD17B13, MBOAT7, PNPLA3,* and *TM6SF2*) with the three NAFLD subtypes showed that only the I148M *PNPLA3* variant was significantly overrepresented in NAFLD patients with subtypes B and C [83]. ALT, AST, and GGT levels were lower in NAFLD patients with subtype A than in patients with subtype C [83]. While in patients with NAFLD subtype C, the relationship between VLDL-TG and VLDL-Apo-B levels and the degree of steatosis was curvilinear, reaching a plateau at steatosis grade S2 (34–66% of hepatocytes contained fat), in patients with subtype A, the levels of VLDL-TG and VLDL-Apo-B were independent of the grade of steatosis from S1 (5–33% of hepatocytes contained fat) to S3 (more than 66% of hepatocytes contained fat) [83]. Taken together, these results support the hypothesis that patients with NAFLD show different VLDL-TG-SR, being lower in patients with subtype A than in patients with subtype B or C.

This hypothesis was confirmed by subtyping a cohort of 20 obese women with NAFLD who had similar age, BMI, and IHTG content but different VLDL-TG-SR [73,83]. It was found that in obese women with NAFLD and subtypes B and C (*n* = 8), VLDL-TG-SR was 2.7-times higher than in obese women with NAFLD and subtype A (*n* = 12). It was also found that the percent of the plasma VLDL-TG pool removed per hour was similar among the three NAFLD subtypes, indicating that the lower levels of VLDL-TG observed in patients with subtype A was mainly due to a reduction in VLDL-TG-SR and not to increased catabolism of the lipoproteins [83]. Taken together, lipidomics has allowed stratification of NAFLD patients, which aligns with different pathophysiological mechanisms. Future directions should further refine the subclassification and identify therapeutic strategy based on the underlying mechanism(s). In addition, the implication of the different NAFLD subtypes on CVD risk warrants further study. Lastly, whether different NAFLD subtypes have different risk for HCC development is of great interest. These efforts will lead us to personalized management of NAFLD patients.

## 3. Conclusions and Future Direction

As shown in this review, hepatic lipid metabolism and 1CM are closely linked. Thus, the deletion of genes encoding the main enzymes involved in 1CM induce hepatic accumulation of fat. In particular, the loss of *Mat1a* induces a drastic reduction in liver SAMe levels, the spontaneous appearance of hepatic steatosis, and its progression to steatohepatitis, fibrosis, and ultimately liver cancer. Of note is the observation that SAMe treatment of *Mat1a*-KO mice reverses liver damage and the finding that, in NAFLD patients, the expression of major enzymes involved in 1CM, including *MAT1A*, is impaired, and the concentration of SAMe in the liver reduced. In addition, in humans, the administration of SAMe has been shown to be safe and beneficial in various liver conditions, such as hepatic cholestasis [85] and alcoholic liver cirrhosis [86]. The possibility of accurately and non-invasively subtyping NAFLD patients provides a unique opportunity to study the effect of SAMe treatment in NAFLD patients with subtype A.

## Figures and Tables

**Table 1.** Noninvasive serum triglyceride biomarkers in NAFLD and NASH. Triglycerides (TG) that participate in the model for the discrimination between NAFLD and normal liver (NL) and/or between NASH and NAFL (simple steatosis) are represented as +.

Individual Notation	TG Involved in NAFLD Diagnostic	TG Involved in NASH Diagnostic	Individual Notation	TG Involved in NAFLD Diagnostic	TG Involved in NASH Diagnostic
TG(44:1)	+	+	TG(52:3)	−	+
TG(46:0)	+	−	TG(52:4)	−	+
TG(48:0)	+	−	TG(53:0)	+	−
TG(48:1)	+	−	TG(53:1)	+	−
TG(48:2)	−	+	TG(53:3)	−	+
TG(49:1)	+	+	TG(54:2)	−	+
TG(50:1)	−	+	TG(54:3)	−	+
TG(50:2)	+	+	TG(54:5) a	+	−
TG(51:1)	−	+	TG(54:5) b	−	+
TG(51:2)	−	+	TG(54:6)	−	+
TG(51:3)	−	+	TG(56:3)	−	+
TG(52:0)	−	+	TG(56:7)	−	+
TG(52:1)	+	−	TG(56:8)	−	+
TG(52:2)	−	+	TG(58:2)	+	−

a and b refer to isomers of the triglyceride TG(54:5) with a mass to charge ratio (*m/z*) = 898.786. TG(54:5) a contains the 18:2, 20:4 and 16:0 acyl chains. TG(54:5) b contains the 18:2, 18:2 and 18:1 acyl chains.

**Table 2.** Predictive metabolic signature for the classification of patients with NAFLD into subtypes A, B, and C. For each subtype, symbols indicate deviations from the mean NAFLD value (+ above mean; − below mean; = almost equal). Each negative / positive symbol represents up to 0.4 standard deviations decrements/increments from the mean.

Lipid Class	Lipid	Subtype		
		A	B	C
Glycerolipids	TG(48:3)	− −	+	+++

Glycerophospholipids	PC(14:0/18:2)	− −	+	+++
	PC(16:0/16:0)	− −	=	+++
	PC(16:0/18:2)	− −	+	+++
	PC(18:0/20:3)	− −	+	+++
	PC(18:3/18:3)	− −	+	+++
	PC(18:0/18:1)	− −	+	+++
	PC(20:0/18:2)	− −	+	+++
	PC(36:3)	− −	+	+++
	PC(37:5)	− −	+	+++

Sphingolipids	SM(32:1)	− −	+	+++
	SM(39:1)	− −	+	+++
